# Thermal Decomposition Mechanism of P(DAC-AM) with Serial Cationicity and Intrinsic Viscosity

**DOI:** 10.3390/polym16111522

**Published:** 2024-05-28

**Authors:** Tingting Chen, Yongji Wang, Yuejun Zhang

**Affiliations:** 1 School of Chemistry and Chemical Engineering, Nanjing University of Science & Technology, Nanjing 210094, China; ctt09011@163.com (T.C.); wyongji2010@126.com (Y.W.); 2 Shaanxi Coal Chemical Industry Technology Research Institute Co., Ltd., Xi’an 710100, China; 3 School of Chemistry and Material Engineering, Chaohu University, Chaohu 238024, China

**Keywords:** P(DAC-AM), thermal decomposition, thermodynamics, kinetics, cationicity, intrinsic viscosity, mechanism

## Abstract

The thermal decomposition of the thermodynamic, kinetic and mechanisms of copolymer P(DAC-AM) samples with serial cationicity and intrinsic viscosity ([*η*]), and the control samples of homopolymer PAM and PDAC, were studied and analyzed using TG, DSC, FTIR. The results of the thermal decomposition thermodynamics confirmed that the thermal decomposition processes of the serial P(DAC-AM) samples and the two control samples could be divided into two stages. It was found that the processes of the copolymer P(DAC-AM) samples were not a simple superposition of those of homopolymers, whose monomers had composed the unit structures of the copolymer, but there were interactions between the two suspension groups. The results of thermal decomposition kinetics showed that the apparent activation energy (*E*) of the thermal decomposition process of all polymer samples had different varying trends in the terms of weight-loss rate (*α*). The reaction order (*n*) of the thermal decomposition of P(DAC-AM) in Stage I and II was close to 1, but in the former and the latter it tended to be 2 and 0.5, respectively. Finally, the thermal decomposition mechanism of copolymer P(DAC-AM) samples was discussed. The above research could not only fill in the knowledge vacancy of the thermal decomposition of the thermodynamic, kinetic and mechanisms of P(DAC-AM), but could also lay a foundation for the study of thermal decomposition mechanisms of the other types of polymers, including cationic polymers.

## 1. Introduction

**Figure 1:**
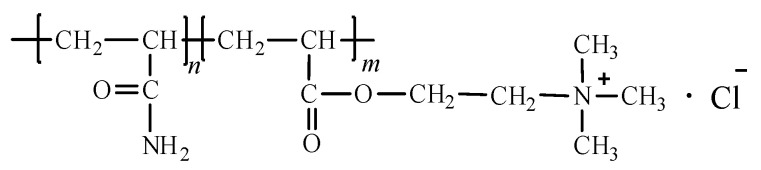
Structure of cationic copolymer P(DAC-AM).

Poly (acryloxyethyltrimethyl ammonium chloride-co-acrylamide) (P(DAC-AM), Figure 1) is a cationic polyacrylamide prepared via the copolymerization of acryloxyethyltrimethyl ammonium chloride (DAC) and acrylamide (AM). Like many other cationic homopolymers and copolymers, it has been widely used in industry and daily life over the years. Especially in recent years, it has also been applied as an electronic chemical and in nanomaterials at a high temperature [1,2]. However, the structure of P(DAC-AM) contains amide, ester and quaternary ammonium groups which easily decompose through heating, so it is urgent to understand the thermal stability of P(DAC-AM).

For polymers, as early as in the 1960s, great attention had been paid to their thermal stability. So far, TG (Thermogravimetry), DSC (Differential Scanning Calorimetry), TG-FTIR-MS (Thermogravimetry-Fourier Transform Infrared Spectroscopy-Mass Spectrometer), and other research methods have been used to study the thermal decomposition process of various quaternary ammonium homopolymers and copolymers [3,4,5,6].

In the 1980s, TG-DSC-GC-MS (Thermogravimetry-Differential Scanning Calorimetry-Gas Chromatography-Mass Spectrometer), GC-FTIR and solid-state C-13 nuclear magnetic resonance (CPMAS) were used to study the thermal decomposition of polyacrylamide (PAM) [6,7,8,9,10,11]. It was shown that in addition to the early weight-loss stage of dehydration, there were only two weight-loss stages due to thermal decomposition, named Stage I and II. In Stage I, the key intermediates were both the cyclic imide formed via the cyclization of suspended chain amide groups and the nitrile group formed via the dehydration of the amide groups, which were obtained through analyzing the structures of gas fragments and solid residues (intermediates). Stage II was mainly the degradation of the cyclic imide structure and main chain to form nitrile, e.g., CO, CO_2_. Due to the limitation of being a single sample, there was neither the correlation of both the thermal decomposition thermodynamic and kinetic parameters with a thermal decomposition mechanism, nor the parametrization of the mechanism.

The thermal decomposition process of quaternary ammonium polymers was studied earlier [12], but its mechanism was involved later [13]. For example, until 2020, Jia X et al. [5,14] used TG-DSC to study the thermal stability of polydimethyldiallyl ammonium chloride (PDMDAAC) samples with three kinds of intrinsic viscosity ([*η*], representing molecular weight). The thermal decomposition thermodynamic and kinetic parameters were obtained. The gas fragments and solid residues of the thermal decomposition in Stage I were analyzed using TG-DSC-MS and FTIR, and the thermal decomposition mechanism was speculated. In Stage I, NH_2_Cl and a small amount of CH_3_Cl fell off to form the pentacyclic alkyl groups or olefin residues. In Stage II, the five-membered ring, olefin residue and main chain broke. This paper was an early report on the thermal stability of homopolymer samples with serial [*η*]. Recently, Fu X Q et al. [15,16] studied the thermal stability of poly(methylacryloxyethyl trimethyl ammonium chloride-co-acrylamide) (P(DMC-AM)) with serial cationicity and [*η*] using TGA-DSC. On the one hand, the thermal decomposition thermodynamic parameters in Stage I and II were measured. Then, the thermal decomposition kinetic parameters were obtained. On the other hand, the gas fragments of the decomposition product of P(DMC-AM) with a cationicity of 50% were analyzed using FTIR and MS, and the thermal decomposition mechanism was deduced combining with the bond order calculation using quantum chemistry. In Stage I, the amide, ester and quaternary ammonium groups began to decompose at the same time, and in turn, the water was removed to generate nitrile, while the alkane–oxygen bond was broken to form acid and the quaternary ammonium group was further decomposed into small molecules. In Stage II, the degradation of nitrile and acid and the decomposition of the C-C skeleton formed more stable residues, which released a large number of small molecules. However, because only one sample with moderate cationicity and [*η*] was tracked in the gas fragments, even the key solid intermediates were not taken into account; the experimental results were greatly limited in supporting the proposed thermal decomposition mechanism of serial samples. In the above literature, they all failed to correlate and explain the thermal decomposition thermodynamic and kinetic parameters obtained with their thermal decomposition mechanism.

For copolymer P(DAC-AM) and its closely related homopolymer PDAC, the study of their thermal decomposition started at the end of the 20th century [17,18,19,20,21,22,23,24]. Since 2006, although the cationicity and [*η*] of the polymerization products P(DAC-AM) reported were different, the thermal stability characterized by the thermodynamic parameters were only for individual samples and even the further mechanism study had not been reported, until recently.

To sum up, the thermal decomposition process of cationic copolymer P(DAC-AM) has two main limitations. On one hand, due to the synthesis level of polyquaternary ammonium salt samples including copolymer P(DAC-AM), there was a lack of samples with serial cationicity and [*η*], which limited the in-depth and comprehensive study of the thermal decomposition process and mechanism. On the other hand, most studies on the thermal decomposition process of polyquaternary ammonium salt samples were often only as an attached property characterization of a single new synthesized sample. Especially for copolymer P(DAC-AM) and its homopolymer, the thermal decomposition process and mechanism of samples with serial cationicity and [*η*] had not been reported so far.

Therefore, concerning samples of the serial copolymer P(DAC-AM) with cationicity of 10~90% and [*η*] of about 5, 10 and 15 dL/g, and with respect to control samples of nonionic homopolymer PAM (homopolymer of monomer AM) and cationic homopolymer PDAC (homopolymer of monomer DAC) with [*η*] about 10 dL/g, the thermal decomposition process and mechanism were systematically studied. Firstly, the thermodynamic parameters were obtained using TG-DSC. Secondly, the thermal decomposition kinetic parameters were calculated according to the thermogravimetric processes. Synchronously, the change rules of thermodynamic and kinetic parameters with the cationicity and [*η*] of P(DAC-AM) samples were investigated, and the preliminary information of the thermal decomposition mechanism was conjectured. Thirdly, based on the literature and the above experimental results, a hypothesis of the possible thermal decomposition mechanism of P(DAC-AM) molecules was proposed. With that, we finally validated the possible intermediates and final products of thermal decomposition. Finally, it was expected that a complete description of the thermal decomposition mechanism of the copolymer P(DAC-AM) with serial cationicity and [*η*] would be obtained, which would be used to explain the experimental parameters and their change phenomena. This is the first time the thermal decomposition mechanism of P(DAC-AM) has been analyzed and discussed in detail.

## 2. Materials and Methods

### 2.1. Raw Material

Using DAC and AM as a monomer and oxidation-reducing agent as an initiator, a series of P(DAC-AM) colloid samples with different cationicity and intrinsic viscosity ([*η*]) were synthesized using temperature programmed in laboratory, and the dry powder samples were obtained after refining via the group method [25]. The specific parameters are shown in Table 1. Where, the cationicity refers to the initial mole fraction of monomer DAC when in synthesis, which ranged from 0% to 100%; the [*η*] represents the molecular weight via a Mark–Houwink equation, which was calculated through measuring the outflow time of the sample in 1 M NaCl solution at (30.0 ± 0.1) °C by using the Ubbelohde viscometer [14].

### 2.2. Measurement Method of Thermal Decomposition Thermodynamic Parameters

The thermogravimetric processes of samples were determined under nitrogen atmosphere using TG (SDTA851E, Zurich, Switzerland) and DSC (METTLER-TOLEDO, Zurich, Switzerland) at the heating rate of 10 K/min [14,15,16]. Then, the thermal decomposition thermodynamic parameters, such as the onset temperature (*T*_s_), the termination temperature (*T*_d_), the decomposition enthalpy (Δ*H*) and the net weightlessness mass fraction of samples after deducting water (*W*) could be obtained.

### 2.3. Measurement Method of Thermal Decomposition Kinetic Parameters

#### 2.3.1. Apparent Activation Energy

The thermogravimetric processes of samples were determined under nitrogen atmosphere using TG at four different heating rates, 5, 10, 20 and 40 K/min [14,15,16]. Then, the apparent activation energy (*E*) of the thermal decomposition kinetic parameters was calculated using Flynn–Wall–Ozawa (FWO) [26,27,28,29,30,31,32,33] method (see Equation (1)).

(1) $$lg\beta =lg\left(\frac{AE}{RG(\alpha )}\right)-2.315-0.4567\frac{E}{RT}$$

where *α*—weight-loss rate, *A*—pre-exponential factor, *β*—heating rate, *R*—universal gas constant, 8.314 J·mol^−1^·K^−1^, *T*—temperature.

#### 2.3.2. Reaction Order and Pre-Exponential Factor

The thermogravimetric processes of samples were determined under nitrogen atmosphere using TG at a heating rate of 10 K/min [14,15,16]. Then, the reaction order (*n*) and pre-exponential factor (*A*) of the thermal decomposition kinetic parameters were calculated using the Coats–Redfern (CR) [34,35] method (see Equations (2) and (3)).

(2) $$\mathrm{For}\;n=1,\;ln\left[\frac{-ln(1-\alpha )}{T^{2}}\right]=ln\left[\frac{AR}{\beta E}\left(1-\frac{2RT}{E}\right)\right]-\frac{E}{RT}$$

(3) $$\mathrm{For}\;n\;\neq \;1,\;ln\left[\frac{1-{\left(1-\alpha \right)}^{1-n}}{T^{2}(1-n)}\right]=ln\left[\frac{AR}{\beta E}\left(1-\frac{2RT}{E}\right)\right]-\frac{E}{RT}$$

### 2.4. Research Method of Thermal Decomposition Mechanism

#### 2.4.1. Quantum Chemistry Calculation for Chain Unit Bond Orders

Using a chain unit from one P(DAC-AM) molecule with representative cationicity as a model (considered as a gaseous molecule, ended with methyl groups in both terminals), Gaussian 09W [15,16] software was adopted with the B3LYP/6-311+G(d, P) basis to calculate the Mulliken bond orders of the target molecule, which was used for sequencing the analysis of weak bonds easily degraded during thermal decomposition under heating conditions.

#### 2.4.2. Intermediate Verification Using FTIR and TG-FTIR/MS

The thermal decomposition processes of P(DAC-AM) samples were measured using TG coupled with FTIR (409PCiS10, Netzsch, Germany, the wavenumber range from 500 cm^−1^ to 4000 cm^−1^) and MS (QMS 403 C Aëolos@, Netzsch, Germany) (TG-FTIR/MS) [15,16]. Each sample was heated from 40 °C to 700 °C at a heating rate of 10 K/min. The gas fragments released during thermal decomposition processes were analyzed using FTIR spectrometer and MS spectra. The solid residues were analyzed using FTIR, which all were used to verify the degradation intermediates in the thermal decomposition processes of P(DAC-AM) samples.

#### 2.4.3. Mechanism Investigation Design

Firstly, combined with the relevant references [17,18,19,20,21,22,23], the thermal decomposition routes or mechanism of P(DAC-AM) molecule was hypothesized based on the analysis of thermal decomposition thermodynamic and kinetic parameters of samples and their changes. Secondly, the quantum chemical calculation of a representative chain unit of P(DAC-AM) molecule was carried out, and the sequencing analysis of the bonds, rated from easily degraded to difficult during thermal decomposition under heating, was conjectured. Thirdly, the net weightlessness rate for each calculation unit on the basis of the cationicity and its change for serial P(DAC-AM) samples was analyzed to speculate the position and the mode of bond breaking. Meanwhile, the gas fragments of thermal decomposition products and the solid residues of the samples in Stage I were analyzed, respectively, using TG-FTIR/MS and FTIR to verify and confirm the structures of the possible intermediates. Finally, based on our hypothesis, a model of the thermal decomposition mechanism of P(DAC-AM) molecules was proposed that fits the observed decomposition processes.

## 3. Results and Discussion

### 3.1. Thermodynamic Parameters and Their Relationship with Molecular Structure

**Figure 2:**
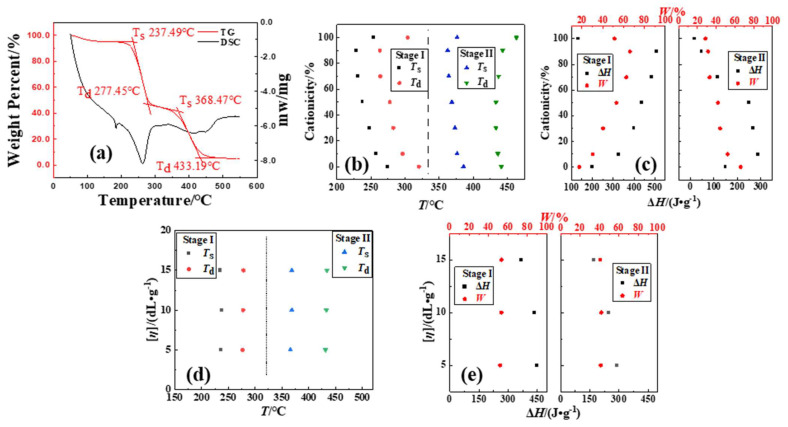
Thermodynamic parameters of P(DAC-AM), PAM and PDAC samples. Notes: (**a**) TG–DSC curve of P(DAC-AM) sample with cationicity of 50% and [*η*] of 10 dL/g; (**b**,**c**) represents *T*_s_, *T*_d_, Δ*H* and *W* of samples with different cationicity in Stages I and II; (**d**,**e**) represents *T*_s_, *T*_d_, Δ*H* and *W* of samples with different [*η*] in Stages I and II.

The TG–DSC curves of the P(DAC-AM) samples with serial cationicity and [*η*], and the control samples PAM and PDAC, were shown in Figure 2a (all TG–DSC curves in Figure S1). The thermogravimetric processes of all samples were very similar in that they could be divided into three stages. In the first weight-loss stage, the TG curve decreased shortly then tended to be gentle because of the evaporation of residual solvents (or moisture, etc.) in the sample [17,18,19,20,21,22,23]. The second and third stages of thermogravimetric processes were the real thermal decomposition stages, which were named as Stage I and II, and their thermal decomposition thermodynamic parameters, such as *T*_s_, *T*_d_, Δ*H* and *W* were obtained and summarized into Figure 2b–e (data in Tables S1 and S2).

(1) Effect of the cationicity

From Figure 2b,c, in Stage I, firstly, when the cationicity of P(DAC-AM) increased from 10% (close to PAM) to 90% (close to PDAC), the proportion of cationic units increased, the *T*_s_ and *T*_d_ decreased and their interval spacing shrank, but the Δ*H* and *W* increased. These changes in *T*_s_ and *T*_d_ indicated that introducing cationic units in the molecular structure of copolymer P(DAC-AM) advanced the thermal decomposition more than that of independently suspended amides or ester-quaternary ammonium salts, meaning that the stability of copolymer P(DAC-AM) decreased with the increase in cationicity. Secondly, with the increase in the cationicity, the share of the thermal effect per unit mass increased, accompanied by the share of weight loss increasing. Since this change coincided with the trend of the increasing proportion of cationic units, it could be inferred that the thermal decomposition of P(DAC-AM) samples in Stage I did not only arise from the fracture of suspended chains, but it is also related to the cationic suspended chains. Finally, compared with the difference in the above obtained data in the *T*_s_, *T*_d_, Δ*H* and *W* of P(DAC-AM) samples with these of the control samples PAM and PDAC, it could be intuitively observed that not only was the decomposition process of the former definitely not a simple superposition of that of the latter two, according to the proportion of their units, but it was also a new and easier thermal decomposition process or mechanism caused by the interaction between the suspended groups, which was able to be created more easily with the increase in the samples’ cationicity.

In Stage II, firstly, when the cationicity of P(DAC-AM) increased from 10% to 90%, *T*_s_ and *T*_d_ slightly moved down and then up, but in a narrow range. Namely, with the increase in cationicity, the initial bonds breaking in reactants in Stage II (decomposition products in Stage I) became slightly easier and the final bonds breaking in these became slightly harder, but they were generally similar. It was indicated that the decomposition processes of reactants were slightly different at this stage due to the slight difference in molecular structures. Secondly, Δ*H* and *W* both decreased with the increase in cationicity, this being the share of thermal effect per unit mass and weight loss of reactants in Stage II decreasing continuously during decomposition. The former indicated that the structures of the reactants were different, while the latter suggested that these reactants corresponded to the change tendency (decrease) of their proportions in the main chain or in the whole molecule at this stage, which further confirmed that the thermal decomposition of P(DAC-AM) sample in Stage II was mainly as a result of the fracture of the main chain in line with the literature. Finally, it was observed that copolymer P(DAC-AM) and homopolymer PAM and PDAC samples had different decomposition processes according to their decomposition thermodynamic parameters in Stage II. However, it was worth mentioning that these parameters of copolymer samples with the lowest and highest cationicity were distinctly close to those situations of PAM and PDAC samples, respectively, which might indicate that they had their own similar decomposition processes in Stage II.

(2) Effect of intrinsic viscosity

From Figure 2d,e, for the different [*η*] of P(DAC-AM) samples with the cationicity of 50% in Stage I and II, the *T*_s_, *T*_d_ and *W* were unchanged with the increase in [*η*], but Δ*H* decreased slightly. It was illustrated that when the chain unit structure was the same, the difficulty of bond breaking was similar with the increase in [*η*], i.e., in the growth of the main chain in either Stage I or II which indicated that the decomposition process might be the same, the [*η*] seemed to have no obvious effect on the thermal decomposition process. However, with the increase in [*η*], the Δ*H* of per unit mass decreased slightly in both stages; it was speculated that the thermal decomposition process might change slightly with the increase in the molecular chain length in Stage I, that is, the thermal effect of the decomposition reaction of the low molecular weight samples with more suspended groups at the edge of chain segment was relatively large. But in Stage II, it might be that the thermal effect required by the break of end groups in the main chain was greater, that is, the larger the [*η*], the fewer the molecular numbers in a same mass and the smaller the share of the terminal groups, which resulted Δ*H* decreasing slightly.

### 3.2. Thermal Decomposition Kinetics

#### 3.2.1. Relationship between Apparent Activation Energy (E) and Reactant Molecular Structures

**Figure 3:**
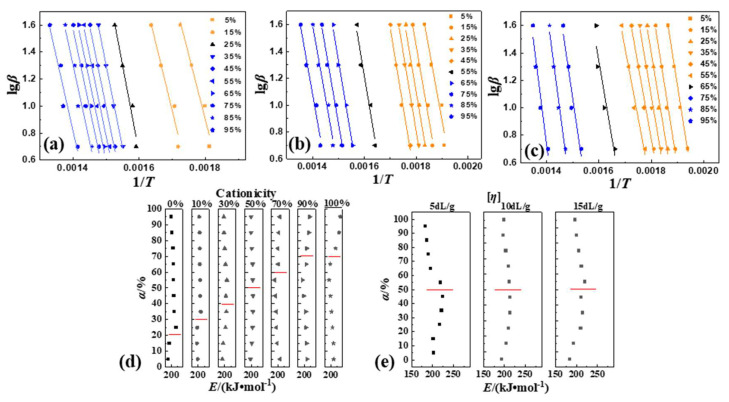
Thermal decomposition kinetic parameters of P(DAC-AM), PAM and PDAC samples using FWO. Notes: (**a**–**c**) Thermal decomposition kinetic curves of P(DAC-AM) samples with cationicity of 0%, 50%, 100% and [*η*] of 10 dL/g; (**d**) represents the change tendency of *E* of samples with different cationicity; (**e**) represents the change tendency of *E* of samples with different [*η*].

Using the plotted curves of lg*β* and 1/*T* with the different *α* (the net weight-loss rate of the sample after deducting water) of P(DAC-AM) samples with serial cationicity and [*η*], the control samples PAM and PDAC were fitted using FWO in Figure 3 (all curves in Figure S2). A good linear dependence between lg*β* and 1/*T* lines were showed for all samples. According to the density of the linear relationship, it could be divided into Stage I (yellow) and II (blue), which was consistent with the situation of thermodynamics. Based on these, the *E* of the thermal decomposition of the samples was calculated and obtained (in Figure 3, data in Tables S3–S5).

(1) *E* of P(DAC-AM) samples with serial cationicity and control samples PAM and PDAC

From Figure 3d, according to the change tendency of the *E* value with *α* for the P(DAC-AM) samples with cationicity ranging from 10% to 90%, alongside the PAM and PDAC samples, the thermal decomposition part after the water weight-loss stage could be divided into two stages for any sample. In Stage I (up the red line), with the increase in the cationicity, the change range of the *E* values increased with the increase in *α*, but the change tendencies of the *E* values were completely different: ① For the cationicity less than 50%, with the increase in cationicity, the *E* values remained unchanged or slightly increased with the increase in *α*, which meant the reaction processes changed and reaction became difficult from easy. ② For the cationicity greater than 50%, with the increase in cationicity, the *E* value decreased significantly with the increase in *α*, which meant the reaction processes changed and the reaction became easy from difficult. ③ For the cationicity of about 50%, there existed a range where *E* was basically unchanged with the increase in *α*, which meant the reaction processes were possibly similar.

However, in Stage II (below the red line), with the increase in the cationicity, *E* decreased gradually with the increase in *α*, but the change tendencies of the *E* values were also different: ① For the cationicity less than 50%, with the increase in cationicity, the *E* values decreased slightly or unchanged with the increase in *α*, which meant the reaction processes changed and the reaction became easy from difficult. ② For the cationicity greater than 50%, with the increase in cationicity, the *E* value increased significantly with the increase in *α*, which meant the reaction processes changed and the reaction became difficult from easy. ③ For the cationicity of about 50%, the *E* values were basically unchanged with the increase in *α*, which meant the reaction processes were possibly also similar. Therefore, the decomposition processes of samples with a different cationicity were different, not only in the change range of the *E* value with *α* in the two different stages, but also in the change tendency of the *E* value with *α* even in the same stage, that is to say, the reaction processes changed with the increase in *α*.

(2) *E* of P(DAC-AM) samples with serial [*η*]

From Figure 3e, the *E* values of the P(DAC-AM) samples with serial [*η*] and a cationicity of 50% changed with the *α*, and there were obviously the faults of the changes or directional contravariant near *α* among 45~55%. And the *E* values of all samples had the same change tendency with *α* in these two stages. Further, it was easy to observe that with the increase in [*η*], the *E* values increased significantly in Stage I and slightly decreased in Stage II when *α* increased. This meant that the thermal decomposition processes of samples with a cationicity of 50% and different [*η*] might be similar, but [*η*] might have still had a little influence on the thermal decomposition mechanism.

#### 3.2.2. Reaction Order and Pre-Exponential Factor of the Thermal Decomposition Reaction

(1) reaction order (*n*)

**Figure 4:**
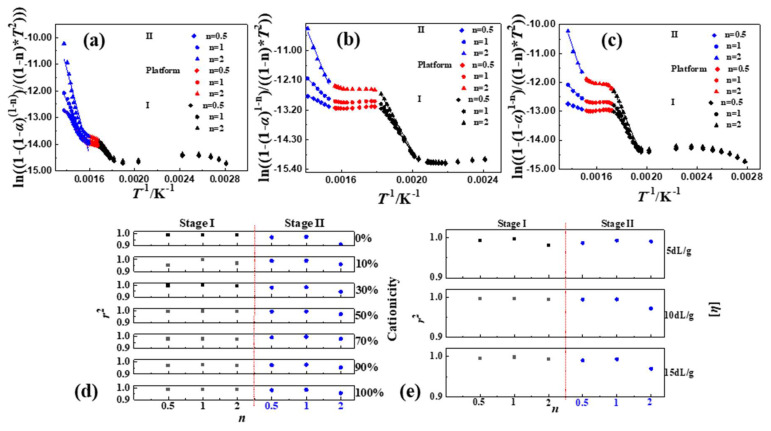
Thermal decomposition kinetic parameters of P(DAC-AM), PAM and PDAC samples using CR. Notes: (**a**–**c**) Thermal decomposition kinetic curves of P(DAC-AM) samples with cationicity of 0%, 50%, 100% and [*η*] of 10 dL/g; (**d**) represents the change tendency of *n* and *r*^2^ of samples with different cationicity; (**e**) represents the change tendency of *n* and *r*^2^ of samples with different [*η*].

Generally, the thermal decomposition reaction of common polymers was conventionally considered as a first order reaction, which meant *n* = 1. Therefore, in this paper, the values of *n* were chosen around 1, i.e., *n* was selected as 0.5, 1 and 2, respectively. Then, the curves in Figure 4 (Figure S3) of the thermal decomposition kinetics of P(DAC-AM) samples with a different cationicity and [*η*], and the control samples PAM and PDAC could be obtained from the linear fitting of $\ln\left(\left(1-{\left(1-\alpha \right)}^{1-n}\right)/\left(\left(1-n\right)T^{2}\right)\right)$ or $\ln\left(-\ln\left(1-\alpha \right)/T^{2}\right)$ with 1/*T*. Firstly, when *α* was under 10%, i.e., 1/*T* was over 0.0020 (the range of temperature was about 50~225 °C), the data points were messy. This was what the evaporation of the moisture or solvent resulted in [19]. Then, when 1/*T* was between 0 and 0.0020 (the temperature was greater than 225 °C), the curves of the thermal decomposition kinetics of all samples were divided into three parts, Stage I, II and Platform, according to the slopes. Finally, based on the linear fitting of each part of the curves, the *n* of the thermal decomposition for each stage could be captured in Figure 4d,e (data in Table S6).

For any sample, the value range of the correlation coefficient (*r*^2^) at *n* = 1 was 0.9756~0.9981 which was larger than that at *n* = 0.5, 0.9516~0.9950 and at *n* = 2, 0.9119~0.9943, in both Stages I and II. This illustrated that $\ln\left(-\ln\left(1-\alpha \right)/T^{2}\right)$ and 1/*T* had a good linear correlation in Stages I and II, when *n* = 1. Therefore, the thermal decomposition reactions of samples in Stages I and II were both close to the one-order reaction, mainly. However, it was not difficult to further observe that in Stage I, when *n* = 2, the values of most *r*^2^ were also higher, so that it was illustrated that in the thermal decomposition process there was some possibility of second-order reaction, which might indicate the reaction between suspended functional groups due to the difficulty for the macromolecular chain segments to move. In Stage II, when *n* = 0.5, the values of most *r*^2^ were also higher, which meant some possibility that the active intermediates of decomposition fragments generated in the reaction process might continue to participate in the subsequent reaction during the thermal decomposition [7].

(2) Pre-exponential factor (*A*)

**Figure 5:**
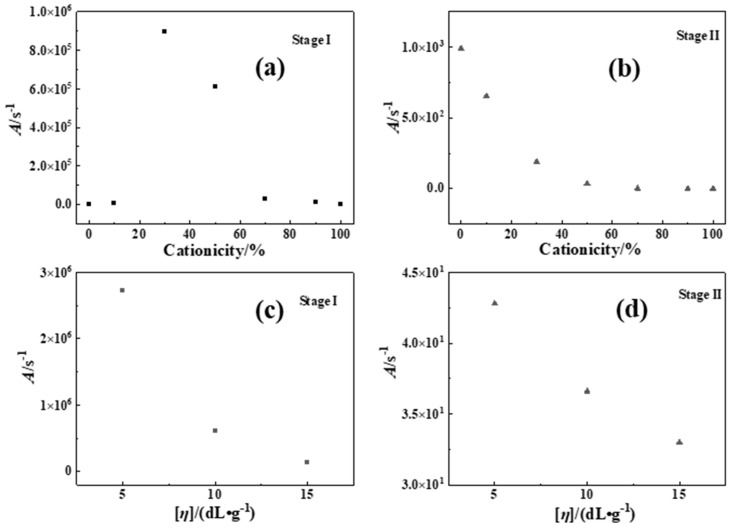
*A* of P(DAC-AM), PAM and PDAC samples using CR. (**a**,**b**) The *A* with different cationicity in Stage I and II; (**c**,**d**) The *A* with different [*η*] in Stage I and II.

According to the experimental results of above (1), it was known that the thermal decomposition of P(DAC-AM) samples with serial cationicity and [*η*] in Stage I and II were mainly close to one-order reaction, which meant *n* = 1. At this time, $\ln\left(-\ln\left(1-\alpha \right)/T^{2}\right)$ and 1/*T* had a good linear correlation. According to the slope of –*E*/*R,* the intercept was $\ln\left(AR/\beta T\right)$, and the *A* could be achieved in Figure 5 (data in Table S7).

On one hand, when the [*η*] of the P(DAC-AM) samples and the control samples PAM and PDAC were the same, with the increase in cationicity in Stage I, the *A* increased first and then stayed in a high value range. It was illustrated that with the increase in cationicity, the collision probability of hanging bonds of samples firstly increased and leveled off in this arrival state. It was suggested that the thermal decomposition types of P(DAC-AM) samples changed with the increase in cationicity, the reaction process after the change was characterized mainly by the collision of reactants (or the functional groups), and the types were similar. In Stage II, the values of all *A* were lower and decreased constantly. It was illustrated that with the increase in cationicity, the cleavage reaction caused by the direct vibration of intramolecular bonds had occupied the main portion. It was speculated that this stage was mainly the vibration fracture of the chemical bonds on the polymer chain. It was suggested that the thermal decomposition processes of samples changed with the increase in cationicity, but the whole was characterized by vibration decomposition and their types were similar. On the other hand, when the cationicity of the P(DAC-AM) samples was the same and the range of [*η*] was 5~15 dL/g, the *A* in Stages I and II changed within a relatively narrow range, indicating that the decomposition processes of the samples were relatively similar, i.e., their reaction types might be similar or even the same.

### 3.3. Thermal Decomposition Mechanism

#### 3.3.1. Hypothesis of Thermal Decomposition Mechanism Model

From the literature [6,11,14,15,16,17,18,19,20,21,22,23,24] and experiment results in Section 3.1 and Section 3.2, a set of hypotheses for a possible mechanism mode of the thermal decomposition is suggested. It was assumed that copolymer P(DAC-AM) sample molecules had several possible thermal decomposition processes in Stage I and II, as follows.

(1) In Stage I, there was an independent decomposition mode of the suspension groups on the chains; the residues were left and the fragments were released after decomposition. The residues were as follows: (a) The amide groups dehydrated independently to form a nitrile group (based on reference [6,11]); (b) the ester groups linked to quaternary ammonium salt were broken to generate unknown products (speculation that needs to be confirmed); (c) only quaternary ammonium salts themselves decomposed or were left to possibly generate carboxylic ester with terminal ethylenic bonds (speculation and based on references [14]), etc. The exfoliated fragments in Stage I further decomposed completely at the same time.
(2) In Stage I, there was an interactive decomposition mode of the suspension groups on chains where the residues were left and the fragments were released after decomposition. The residues were as follows: (a) The reaction between amide groups formed the cyclic imides (based on reference [6,11] and kinetic parameters); (b) the reaction between an ester group linked to quaternary ammonium salt and an amide group formed an unknown product (based on thermodynamic and kinetic parameters); (c) the reaction between ester groups linked to quaternary ammonium salts themselves formed unknown products (based on thermodynamic and kinetic parameters). Further, the exfoliated fragments in Stage I decomposed completely at the same time.
(3) In Stage II, there was a residue decomposition mode: A variety of structural residues formed in Stage I that were the solid intermediates that acted as the reactants in Stage II and continued to decompose.

#### 3.3.2. Theoretical Simulation and Experimental Verification

##### Bond Order Calculation using Quantum Chemistry

**Figure 6:**
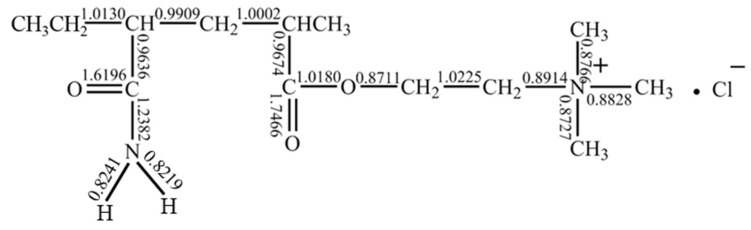
Distribution of molecular bond order of P(DAC-AM).

The chain segment of the polymer P(DAC-AM) molecule with the simplest unit combination structure, namely the sample molecule with a cationicity of 50% (one unit for positive and one unit for non-ionic, which might also approximately represent the two units of both the PAM and PDAC chain simultaneously), was selected to obtain the Mulliken bond order of each bond in the unit structure of the P(DAC-AM) molecule using quantum chemical calculation. The results are shown in Figure 6.

On one hand, for non-ionic units, the N-H bond had the smallest bond order, which meant it was easiest to break or to react with other groups. It was speculated that amide groups could attack adjacent amide groups, and NH_3_ and H_2_O as small molecules mainly dropped off to generate cyclic imide and nitrile structures [32,33,34,35], or, further, they might attack the carbonyl carbon on the adjacent cationic unit and the -OCH_2_CH_2_N^+^(CH_3_)_3_Cl^−^ fragment that dropped off to form a cyclic imide structure. On the other hand, for the cationic units, the bond order increased in turn as the alkoxy C-O bond of ester group (0.8711) < methyl C-N bond of quaternary ammonium group (0.8727–0.8828) < C-N bond of quaternary ammonium salt and alkyl group (0.8914) < C-C bond of carbonyl group and main chain (0.9636–0.9674) < C-C bond of main chain, which meant the alkoxy C-O bond of the ester group was most likely to break, and then the methyl group on the quaternary ammonium salt itself broke.

Thus, it was speculated that the alkyl–oxygen bond of the ester group was easiest to break on the cationic unit. And then during the independent thermal decomposition, the -CH_2_CH_2_N^+^(CH_3_)_3_Cl^−^ fragment mainly fell off to form carboxylic acid as a residue; it was further speculated that alternatively carboxylic anhydride was formed by the reaction of the carboxylic acid group attacking the adjacent carboxylic acid (ester) group following the time of its fracture, and the -CH_2_CH_2_N^+^(CH_3_)_3_Cl^−^ and -OCH_2_CH_2_N^+^(CH_3_)_3_Cl^−^ fragments mainly fell off.

##### Weight Loss Comparison and Analysis

According to the proposed mechanism and the theoretically derived bond orders, (1) the fragments that might fall off via their independently degradations based on the model mentioned were H_2_O, -CH_2_CH_2_N^+^(CH_3_)_3_Cl^−^ and -N^+^(CH_3_)_3_Cl^−^. (2) The fragments that might fall off via the possibly interactive degradations based on the model mentioned were NH_3_, -OCH_2_CH_2_N^+^(CH_3_)_3_Cl^−^ and -CH_2_CH_2_N^+^(CH_3_)_3_Cl^−^ combined with -OCH_2_CH_2_N^+^(CH_3_)_3_Cl^−^. According to these exfoliated fragments and modes that these hanging bonds degraded independently or through interaction, the corresponding theoretical weight loss of the P(DAC-AM) molecule was obtained only via one of the all above modes. The theoretical weight loss under these different modes and the actual weight loss (i.e., *W*/%) obtained from the thermal decomposition thermodynamic measurement of the sample is compiled in Table 2.

For homopolymers, according to the literature [6,7,8,9,10,32,33,34,35], PAM in Stage I was mainly the exfoliated small molecules NH_3_ and H_2_O, and according to the actual weight loss 17.48%, it could be calculated that the proportions of exfoliated NH_3_ and H_2_O were 59% and 41% (mole ratio, listed in Table 3), respectively. For PDAC, according to the comparison of the theoretical weight loss of each exfoliated structure with the practical one 51.31%, the PDAC in Stage I should shed mainly -N^+^(CH_3_)_3_Cl^−^ (48.84%), accounting for 87% of PDAC weight, and only a few other fragments were exfoliated necessarily.

For copolymer P(DAC-AM) with a cationicity of 10~90%, on one hand, we argued that the thermal decomposition processes or their fragments were absolutely not a simple superposition of the independent decomposition processes of non-ionic and cationic suspension groups on chains, which was proved again using the contents in Table 2. On the other hand, combined with the analysis of experimental results and the bond order determination, it could be known that in the thermal decomposition process, the suspension group might not only break and degrade independently, but also include the correlated degradation between various forms of groups. Therefore, based on the different cationicity of copolymers, it is not difficult to see the following:

(1) When the cationicity was around 50%, the theoretical fall in weight loss of the -OCH_2_CH_2_N^+^(CH_3_)_3_Cl^−^ fragment was very close to the actual one. It was estimated that the thermal decomposition in Stage I was mainly due to when the amides attacked ester groups to form cyclic imide, and the -OCH_2_CH_2_N^+^(CH_3_)_3_Cl^−^ fell off. Furthermore, it was inferred that the interaction between non-ionic and cationic units happened dominantly; in other words, the interactive degradation form was preferred. Therefore, it could be inferred that this mechanism should be one of most common degradation reaction forms in Stage I of the thermal decomposition process of P(DAC-AM) with a cationicity of 10~90%, and its proportion would of course depend on the unit proportion of an amide with an ester group in the consecutive position in whole chain units, which firstly increased then decreased with the increase in cationicity.
(2) When the cationicity was less than 50%, the molecular structure of the samples was dominated by non-ionic units. According to the above (1), the interaction between non-ions and cations was preferred in the degradation process so that the reaction process of the remaining non-ionic units was similar to that of PAM. That is, it could be speculated that the thermal decomposition in Stage I was when the NH_3_, H_2_O and -OCH_2_CH_2_N^+^(CH_3_)_3_Cl^−^ fell off, i.e., when the independent reaction of amide, the reaction between the amide groups and the new reaction of amide groups attacking their neighbor ester groups to generate cyclic imide mainly occurred.
(3) When the cationicity was greater than 50%, the molecular structure of the samples started to be gradually dominated by cationic units. According to above (1), the interaction between non-ions and cations was preferred in the degradation process so that the reaction process of the remaining cationic units might be similar to that of PDAC. However, due to the great difference between the theoretical and actual weight loss once supposed in this reaction form, it was speculated that with the increase in cationic units, the new interaction between cation units might exist. That was, except for shedding the fragments -OCH_2_CH_2_N^+^(CH_3_)_3_Cl^−^, the fragments -OCH_2_CH_2_N^+^(CH_3_)_3_Cl^−^ with -CH_2_CH_2_N^+^(CH_3_)_3_Cl^−^ might fall off at the same time. Furthermore, it was speculated that the thermal decomposition in Stage I might be the interaction both between amide and ester groups to generate cyclic imide and between ester groups to generate anhydride [9]. Especially, for the control sample PDAC with a cationicity of 100%, from the weight loss of fragments and the fact of no longer existing due to the deficiency of the corresponding reaction caused by amide groups, it was speculated that except for shedding the fragment -N^+^(CH_3_)_3_Cl^−^, there was still the -OCH_2_CH_2_N^+^(CH_3_)_3_Cl^−^ with -CH_2_CH_2_N^+^(CH_3_)_3_Cl^−^ that fell off at the same time. Thus, it was speculated that the decomposition process comprised quaternary ammonium salt shedding accompanied by a small amount of interaction between ester groups, leaving corresponding vinyl acylate and anhydride attached on the main chain as residue intermediates.

For the thermal decomposition of PAM, PDAC and P(DAC-AM) samples in Stage I, according to the above decomposition process and based on the distribution of cationicity, the best combination matching was performed through prioritizing interactions. Therefore, their respective proportions (Table 3, in parentheses) and the comparison between the actual and theoretical weight loss calculated according to the combination in the thermal decomposition processes in Stage I of all samples are shown in Table 3. The relative error between the theoretical and actual weight loss based on the distribution of cationicity and the optimal fragment combination by a rule of interaction priority was shown within 10%, which indicated the rationality of the above speculation on the structure and combination of exfoliated fragments in the thermal decomposition processes of P(DAC-AM) in Stage I fairly well. At the same time, it also laid a foundation for the identification of the fragments in Stage II.

##### FTIR/MS Tracking Analysis of Decomposed Fragments

A P(DAC-AM) sample with a cationicity of 50% and the simplest chain segment structure was selected, and the TG-FTIR/MS method was used to obtain their FTIR spectra of gas fragments generated in the thermal decomposition and then their mass-to-charge ratio (*m*/*e*) from molecular ion peaks after electron bombardment, which was used to verify the hypothesis and structure of the fracture fragments mentioned.

From FTIR and MS data (Table 4), in Stage I, the characteristic absorption peaks of the C-Cl and N-C of tertiary amine were detected at 709 and 1367 cm^−1^. It was suggested that the fragment with a *m*/*e* of 50 and 52 might be CH_3_Cl, and that with a *m*/*e* of 59 might be N(CH_3_)_3_, which only appeared in Stage I. It was confirmed that the quaternary ammonium group of cations fell off and further decomposed in Stage I. At the same time, the characteristic absorption peak of primary alcohol C-OH was detected at 1033–1053 cm^−1^, so it was presumed that the fragment with a *m*/*e* of 47 might be CH_3_CH_2_OH. It was confirmed that the P(DAC-AM) sample with a cationicity of 50% might interact with non-ions and cations in Stage I, and the exfoliated fragment was HOCH_2_CH_2_N^+^(CH_3_)_3_Cl^−^ accompanied by the further exhalation of the quaternary ammonium salt.

In Stages I and II, the stretching vibration absorption peaks of the N-H and O-H of the fragments were detected at 3300~4000 cm^−1^, and the characteristic absorption peaks of H_2_O (*m*/*e*: 18) and NH_3_ (*m*/*e*: 17) were detected at 1626 and 1158 cm^−1^, which indicated that the amide group dehydrated and shed ammonia in Stage I to form possible nitrile groups and ring imide structures of solid residues (intermediates), respectively. Meanwhile, the characteristic absorption peaks of C≡N were detected at 2383~2305 and 2180~2105 cm^−1^. It might be the fragment CH_3_CN (*m*/*e*: 41), which only appeared in Stage II. Thus, it was confirmed from another viewpoint that in the fracture modes of amide groups in Stage I, the nitrile group (less) formation via dehydration and the imide intermediate generation (mainly) from the interaction between the two suspension groups on chains existed, which was the reason for a small number of possible CH_3_CN fragments being detected in Stage II.

In Stage II, it is noteworthy that a significant amount of the oxygen-containing small molecules and hydrocarbon fragments could be observed, in addition to those degradation fragments (various hydrocarbon molecules) derived from carbon chain skeletons. For example, the characteristic absorption peak of aldehyde C=O was detected at 1735 cm^−1^, and the characteristic absorption peaks of CO_2_ were detected at 2383~2305 and 671 cm^−1^. It was presumed that the fragment with a *m*/*e* of 44 might be CH_3_CHO and CO_2_. Therefore, it was speculated that there were a small number of other oxygen-containing functional groups in the initial reactants of Stage II, i.e., the solid residues of Stage I, which might come from the anhydride intermediate generated through the interaction between the neighbor ester groups of cationic units. In fact, the thermal decomposition of residues was the degradation of the carbon skeleton and the residue intermediates.

Therefore, it was confirmed that for P(DAC-AM) with the cationicity of 50%, the interaction between non-ionic and cationic suspension groups dominated Stage I. The fragment HOCH_2_CH_2_N^+^(CH_3_)_3_Cl^−^ fell off and was further decomposed into small molecules. However, due to the inhomogeneity in the linking process of the two kinds of monomers, i.e., the block polymerization in the copolymerization process existed consequently, it could not be ignored that both the dehydration and deamination of the non-ionic units to form nitrile and cyclic imide structures and the reaction between the cation units to form anhydride could occur simultaneously.

##### Characterization and Analysis of Solid Residues

**Figure 7:**
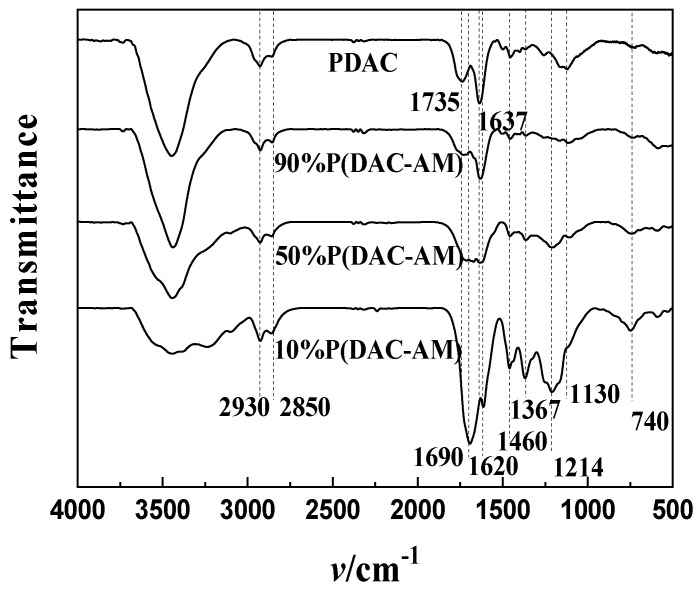
FTIR spectra of the residues after the end of Stage I.

In order to further verify the structural speculation of solid residues (intermediates) in Stage I, and to verify the prediction of thermal decomposition fragments in the Section Weight Loss Comparison and Analysis, the FTIR analysis was performed on the residues after the thermal decomposition of the P(DAC-AM) samples with a cationicity of 10%, 50% and 90% and the PDAC samples in Stage I, i.e., the reactants in Stage II; their FTIR spectra are shown in Figure 7.

From Figure 7, it could be clearly seen that in the residue spectra of both the P(DAC-AM) samples and PDAC samples, firstly, for all of samples, the stretching vibration absorption peak of N-H was detected at 3300~4000 cm^−1^, and the stretching vibration absorption peaks of the saturated carbohydrate C-H were detected at 2930 and 2850 cm^−1^. Particularly, the stretching vibration absorption peak of C-N of imide was detected at a 1460 cm^−1^, and the deformation vibration of the ring was detected at 740 cm^−1^, all of which indicated the existence of a cyclic imide structure. Furthermore, since the stretching vibration absorption peak of C-N of imide was detected around 1460 cm^−1^ and then gradually decreased to almost disappear with the increase in cationicity, it was speculated that the cyclic imide structure declined or was replaced by another cyclic acyl structure, such as a possible structure somewhat like a cyclic ester or anhydride.

Secondly, the residue spectrograms of both the P(DAC-AM) with a cationicity of 10% and 50% samples and the P(DAC-AM) with a cationicity of 90% and PDAC samples were considered due to their systematic mutual comparison, respectively. It was not hard to discover through the contrast directly, on the one hand, that the stretching vibration absorption peaks of C=O on the imide were detected at about 1690 and 1620 cm^−1^ of the former samples, which indicated that there was a diacetyl imide structure in the residues. This further confirmed the possibility that the H atom of the amide group on the non-ionic unit attacked the carbonyl carbon on the cationic unit to form the cyclic imide structure, resulting in the HOCH_2_CH_2_N^+^(CH_3_)_3_Cl^−^ shedding. On the other hand, in the spectrograms of the residues of the latter samples, there was no obvious absorption peak of the imide, but only the absorption peak of the carboxylic ester was detected at about 1735 cm^−1^, and the stretching vibration absorption peaks of the C-O-C of anhydride were detected at 1130 and 1214 cm^−1^. It also confirmed that the cationic units interacted with each other to form cyclic anhydride resulting in -OCH_2_CH_2_N^+^(CH_3_)_3_Cl^−^ and -CH_2_CH_2_N^+^(CH_3_)_3_Cl^−^ shedding.

Finally, in the residue spectra of both P(DAC-AM) with a cationicity of 90% and PDAC samples in Stage I, the stretching vibration absorption peak of C=C was detected at 1637 cm^−1^, which indicated that in both the copolymer with a high cationicity and the cationic homopolymer the exfoliated fragments of HN^+^(CH_3_)_3_Cl^−^ existed and the vinyl acrylate remained. This confirmed the hypothesized fracture mode of the quaternary ammonium group.

Therefore, it could be seen from all the above FTIR spectrogram analysis results that the characteristic groups on the residues from all samples in Stage I were consistent with the recognitions obtained in the Section Weight Loss Comparison and Analysis, which commonly verified the corresponding mechanism hypothesis.

#### 3.3.3. Description of Thermal Decomposition Mechanism

##### Mechanism Description

Based on the hypothesis and validation analysis of the thermal decomposition mechanism of cationic copolymer P(DAC-AM) (10–90%) samples and control samples PAM (0%) and PDAC (100%), the thermal decomposition mechanism could be obtained and described as follows.

First at all, the thermal decomposition process of all polymer samples after solvent evaporation was generally divided into two stages, Stage I and II, respectively, corresponding to the thermal decomposition of suspended groups on the suspended chain in Stage I and the further decomposition of the residues from Stage I, i.e., intermediates used as the reactants in Stage II. Then, the thermal decomposition process or mechanism of each polymer sample mainly changed with the content of cationic units on the molecular chain, which is cationicity. The thermal decomposition processes or mechanism of the samples with the same cationicity and different [*η*] were almost similar. That was to say, there were three kinds of core or main reaction processes or mechanisms of thermal decomposition with the changed cationicity in Stage I and II:

(1) When the cationicity was 0%, the degradation in the suspended chain of non-ionic units mainly happened, where both the amide groups were independently dehydrated to form the nitrile group and the neighbor amide groups through deamination were closed loop to form cyclic imide residues. Then, the nitrile and cyclic imide residues were further degraded along with the main chain.
(2) When the cationicity was about 50%, the suspended chain was mainly degraded via the interaction between non-ionic and cationic units to form cyclic imide, and HOCH_2_CH_2_N^+^(CH_3_)_3_Cl^−^ fell off. Then, the cyclic imide was further degraded along with the main chain.
(3) When the cationicity was 100%, the suspended chain was mainly degraded by both the reaction between neighbor cationic units themselves and the independent decomposition of the cationic units. The former discharged -OCH_2_CH_2_N^+^(CH_3_)_3_Cl^−^ and -CH_2_CH_2_N^+^(CH_3_)_3_Cl^−^ fragments to generate cyclic anhydride synchronously, while the latter only removed quaternary ammonium salts, leaving vinyl acrylate residues. Then, the cyclic anhydride and vinyl acrylate were further degraded along with the main chain.

**Figure 8:**
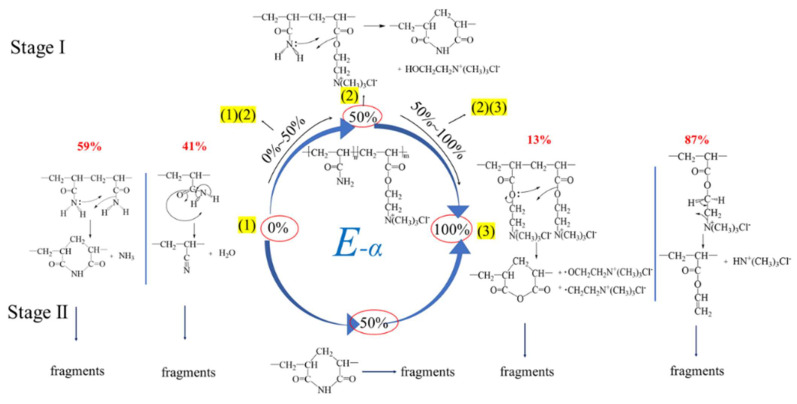
Mechanism of thermal decomposition of P(DAC-AM) in Stage I and Ⅱ.

Therefore, for the cationic copolymer P(DAC-AM) samples, when the cationicity was within 10~50% or 50~100%, any one of their thermal decomposition processes or mechanisms were the combination of either the above core or main reaction processes (1) and (2) or (2) and (3), according to the cationicity. The above description could be expressed in Figure 8.

##### Mechanism Explanation of Thermal Decomposition Thermodynamics Parameters

According to the proposed thermal decomposition mechanism of P(DAC-AM), it could be used to interpret the thermodynamic parameters measured in thermal decomposition processes and their change regulations in the experiment more comprehensively.

(1) *T*_s_ and its change

The reasons for these might be, on the one hand, based on the above decomposition mechanism in the Section Mechanism Description and Figure 8; with the cationicity increasing, the degradation reaction changed from the reaction-only amide groups themselves (0% cationicity, PAM) to form mainly the nitriles and cyclic imides, then from the reaction of amides with esters (10~50% cationicity, copolymers) to form the cyclic imides, then they changed even more from the reaction of the ester groups themselves (50~90% cationicity, copolymers) mainly to form cyclic anhydrides and finally they changed from the reaction only of esters with quaternary ammonium salts mainly (100% cationicity) to form vinyl acrylate. Accompanied with the increase in cationicity units in the copolymer P(DAC-AM) chain, the new reaction mechanisms gradually occurred, the starting degradation of polymers became easier, and *T*_s_ decreased more and more (Figure 2b).

On the other hand, it could be seen from the decomposition mechanism (Figure 8 and Table 3) that the portions of the independent degradation of suspended groups on chains in the two homopolymers were obviously more or close to one half of interactive degradation, indicating that they were the easier ones in their own homopolymers and a little difficult in comparison with their neighbor copolymers with similar cationicity, because of the hybrid function when the different suspended groups existed together. So, the *T*_s_ of homopolymers were higher. Herein, since it is more difficult for the reaction of amides dropping off water to form nitrile than for the reaction of esters dropping off quaternary ammonium salt to form the vinyl acrylate, the *T*_s_ of PAM was higher in the control samples.

Furthermore, for the thermal decomposition in Stage II, the difference in *T*_s_ between homopolymers and copolymers and among copolymers with a different cationicity was significantly reduced, because the cyclic imide and cyclic anhydride had become the dominant intermediates, and the former was clearly observed to be more stable. In addition, it was particularly interesting that the *T*_d_ of almost all the sample molecules (except for the PDAC with cationicity of 100%) was very similar, suggesting that the reaction types, i.e., the chemical bonds broken at the last stage of thermal decomposition should be mainly broken carbon–carbon bonds and have their own similarities (Figure 2b).

(2) Δ*H*, *W* and their change

From Figure 2c, it could be seen that the Δ*H* and *W* in Stage I greatly increased with the increase in cationicity from 0% to 90%. According to the mechanism description in Figure 8, this resulted from the change of reaction mechanism with the change in cationicity. Due to the increase in exfoliated fragment molecules, the *W* increased and the energy costs of exfoliated fragments and subsequent decomposition increased, i.e., Δ*H* increased synchronously. For PDAC with a cationicity of 100%, due to the different decomposition mechanisms, the exfoliated fragments were smaller and the Δ*H* and *W* also decreased, respectively.

Furthermore, the main reason for the great decrease in the Δ*H* and *W* with the increase in polymer cationicity from 10% to 100% in Stage II of the thermal decomposition was due to the gradual decrease in residue mass portions in Stage I, resulting in the energy costs of residue decomposition decreasing. However, it could not be ignored that the contribution of changes in residue structures to the Δ*H* decaying, such as from cyclic imides mainly to anhydrides and then mainly to vinyl acrylate (in Figure 8) resulted in energy costs decreasing. Furthermore, PAM with a cationicity of 0% had a few lower Δ*H* than what its neighbor copolymer had, because of different decomposition mechanisms in Stage I and residue structures.

##### Mechanism Explanation of Thermal Decomposition Kinetic Parameters

(1) Stage I of thermal decomposition

The initial apparent activation energy in Stage I and II (*E*_(I)_ and *E*_(II)_) of the polymer samples with a different cationicity in Figure 3d were summarized as shown in Table 5. On the one hand, with the increase in cationicity, the *E*_(I)_ required for the initial decomposition had an obvious increasing tendency to increase. This could be due to the following: Firstly, due to the minimal steric hindrance, the collision probability of amide functional groups for the reaction generating cyclic imides and nitriles was bigger (the larger value of *A*, 10^2^~10^5^ magnitude, *n* = 1~2), and the reaction was easy, so *E*_(I)_ was low (although the *T*_s_ of samples with cationicity of 0% was high). Secondly, the steric hindrance of the reaction of amide groups attacking ester groups with large groups to generate cyclic imide increased, the collision probability decreased, and the reaction became slightly difficult, so the *E*_(I)_ increased. Finally, the reaction in which the simultaneous or subsequent carboxylic acid group attacked the adjacent ester groups to form cyclic anhydrides was more difficult to process due to the high steric hindrance and obvious collision obstruction.

On the other hand, just as previously observed in Figure 3d, the *E* in Stage I for each of all the polymer samples changed in a different degree with the *α* increasing, which indicated that the reaction mechanism changed in Stage I. The *E* of the samples with cationicity increased by less than 50% and more than 50% decreased. According to the decomposition mechanism described, the reasons should be as follows: For the samples with low cationicity (since 0%), the main decomposition carried on through the interaction of suspended amide groups to form cyclic imides and the independent dehydration of themselves to form nitriles. Here, the former was easily carried out due to the stable product and low *E* with the latter being carried out with difficultly, so the *E* of these samples increased with the *α* increasing. Then, with the increase in cationicity, the interaction of amide groups was gradually replaced by the amide groups attacking the neighbor ester groups. Although the *E* of the interaction forming the cyclic increased due to the difficulty of the mutual collision, the reaction was easily carried out due to the stable cyclic product and lower *E*. Finally, for the samples with a cationicity of more than 50%, especially the samples with high cationicity, the interaction between the cationic units to form the cyclic anhydrides as products could occur via a pre-step where the esters decomposed to form carboxylate radicals which then attacked their neighbor carbonyl (group) carbon atoms. In this process, the decomposition *E* of the formation of carboxylate radicals became larger, so the *E* decreased while *α* increased.

(2) Stage II of thermal decomposition

With the increase in cationicity, the *E*_(II)_ required for the initial decomposition in Stage II tended to decrease significantly which is a sharp contrast. According to the above description of the thermal decomposition mechanism, the reasons should be as follows: The structure stability of the intermediate formed in Stage I, namely the molecule (chain unit) of the reactant in Stage II (the extremely small value of *A*, 10^−1^~10^2^ magnitude, *n* = 1~0.5), determined the ease of the thermal decomposition reaction, that is, the value of the *E*_(II)_ of the reaction. The thermal stability of cyclic imide was generally considered to be better than that of cyclic anhydride, that is, the mass fraction of cyclic imide (samples with low cationicity) was larger, the *E*_(II)_ was higher, and the *E*_(II)_ decreased with the reduction in the cyclic imide share or the increase in the cyclic anhydride share.

It was particularly interesting and noteworthy that the *E* required for the final decomposition in Stage II tended to rise sharply as the cationicity increased, especially in the samples with a high cationicity such as 100% which formed the intermediates like anhydrides and mainly vinyl acrylate as the reactants in Stage II. According to the above (1) and Figure 2b, for the samples with a high cationicity, the *T*_d_ at the end of the thermal decomposition also had the same tendency. The reasons might be that the new unknown intermediate structures with a better thermal stability and more difficulty in decompositions were generated after starting the decomposition process in Stage II of the samples with a high caitonicity and continued to decompose even further.

## 4. Conclusions

(1) The thermal decomposition processes of the P(DAC-AM), PAM and PDAC samples could be divided into two stages. The ranges of *T*_s_, *T*_d_, Δ*H* and *W* in Stage I were obtained and are as follows: 228.48~274.35 °C, 262.99~320.13 °C, 130.91~507.42 J/g and 17.48~66.11%, the ranges of *T*_s_, *T*_d_, Δ*H* and *W* in Stage II were obtained and are as follows: 362.42~385.61 °C, 431.36~463.20 °C, 17.19~288.34 J/g and 28.47~66.02%. It was found that the decomposition process of copolymers was not a simple superposition of that of their homopolymers and there were interactions between the suspension groups on copolymer main chains. The decomposition processes of P(DAC-AM) were different with the different cationicity. However, the effect of [*η*] on the thermal decomposition processes was small.
(2) The *E* values of the thermal decomposition of the P(DAC-AM), PAM and PDAC samples ranged from 173.56 to 274.08 kJ/mol. It was found that the *E* values or their changes could be divided into two ranges with the change in *α*, which corresponded to two stages of thermal decomposition thermodynamics. For samples with different cationicity, the ranges of these two stages were different, indicating that the decomposition processes varied with the cationicity. The *E* value of the same sample obviously increased or decreased in the respective *α* range, which meant that the decomposition processes changed continuously with the *α*. However, the *E* values of P(DAC-AM) with a cationicity of 50% were very close to each other in the two ranges of *α*, respectively, which indicated that the decomposition processes were basically the same for both ranges. At the same time, the decomposition processes were slightly different with a different [*η*], when with the same cationicity.
(3) The *n* of thermal decomposition of the P(DAC-AM), PAM and PDAC samples in Stage I was calculated to be between the 1 and 2 order reaction, and the *A* value of this stage had the higher order of magnitude of 10^2^~10^5^ indicating that the thermal decomposition stage was mainly the fracture reaction of the sample molecules, but there were collisions between the molecules or functional groups and the collisions were the most intense in the sample with a cationicity of 50%. The *n* of Stage II was between 0.5 and 1 order reaction and the *A* value of this stage had the lower order of magnitude of 10^−1^~10^2^, indicating that the thermal decomposition of the reactants (products in Stage I) in this stage was dominated by bond fracture and there were a chain reaction and transfer effect of degrading intermediate fragments, with this effect increasing with the increase in cationicity.
(4) It was discovered that there were three novel mechanisms of thermal decomposition for all polymer samples, each with a different cationicity. In Stage I, the suspended amide groups attacked the neighboring ester groups to form the cyclic imide intermediates, accompanied by the dropping off of hydroxyalkyl quaternary ammonium salts and their further decomposition. The suspended ester groups attacked adjacent ester groups to generate cyclic anhydride intermediates, and the hydroxyalkyl quaternary ammonium salts and alkyl quaternary ammonium salts were detached and further decomposed. The suspended ester groups removed the quaternary ammonium salts to form vinyl acrylate intermediates. In Stage II, all the intermediates along with the backbone involved were further decomposed.
(5) Based on the literature and our results, the new thermal decomposition processes of the P(DAC-AM), PAM and PDAC samples were proposed and rationalized. The mechanism description could systematically, comprehensively and reasonably explain the experimental parameters measured in this study and their variation rules. The above results could provide guidance on the application of P(DAC-AM) in electronic chemicals and nanomaterials at a high temperature.

## Figures and Tables

**Table 1:** Parameters of P(DAC-AM), PAM and PDAC samples.

No.	Cationicity/%	Simplified Representation [*η*]/(dL·g^−1^)
1	0 ^a^	10
2	10/30/50/70/90	10
3	50	5/10/15
4	100 ^b^	10

Notes: ^a^ Representing nonionic homopolymer PAM. ^b^ Representing cationic homopolymer PDAC.

**Table 2:** Theoretical and actual weight loss of P(DAC-AM), PAM and PDAC samples in Stage I.

Cationicity/%	Theoretical Weight Loss in Stage I (Only Fragment)/%						Actual Weight Loss in Stage I/%
	H_2_O	NH_3_^a^	-OR ^b^	-OR and -R ^c^	-R ^d^	-N^+^(CH_3_)_3_Cl^−^	
0	25.35	11.97	-	-	-	-	17.48
10	19.46	9.19	16.64	15.68	14.71	11.35	30.26
30	11.69	5.52	38.56	36.33	34.11	26.31	40.27
50	6.81	3.21	52.36	49.34	46.31	35.73	53.10
70	3.44	1.63	26.51	58.28	54.70	42.20	62.55
90	0.99	0.47	7.64	64.80	60.83	46.92	66.11
100	-	-	-	67.44	63.31	48.84	51.31

Note: ^a^ NH_3_ was produced from the interaction between the nonionic and nonionic units; ^b^ -OR represents -OCH_2_CH_2_N^+^(CH_3_)_3_Cl^−^ and was produced from the interaction between the nonionic and cationic units; ^c^ -OR and -R represent -OCH_2_CH_2_N^+^(CH_3_)_3_Cl^−^ and -CH_2_CH_2_N^+^(CH_3_)_3_Cl^−^ which were produced from the interaction between the cationic and cationic units; ^d^ -R represents -CH_2_CH_2_N^+^(CH_3_)_3_Cl^−^.

**Table 3:** Comparison between the actual and theoretical weight loss.

Content		Proportion						
**Cationicity/%**		**0**	**10**	**30**	**50**	**70**	**90**	**100**
Exfoliated structures	NH_3_	√(59%)	√(47%)	√(24%)				
	H_2_O	√(41%)	√(33%)	√(16%)				
	-OR ^a^		√(20%)	√(60%)	√(100%)	√(60%)	√(20%)	
	-OR and -R ^b^					√(40%)	√(80%)	√(13%)
	-N^+^(CH_3_)_3_Cl^−^							√(87%)
Weight loss in Stage I	Theoretical/%	17.48	28.57	43.13	52.36	59.81	65.24	51.31
	Actual/%	17.48	30.26	40.27	53.10	62.55	66.11	51.31

Note: ^a^ -OR represents -OCH_2_CH_2_N^+^(CH_3_)_3_Cl^−^; ^b^ -OR and -R represent -OCH_2_CH_2_N^+^(CH_3_)_3_Cl^−^ and -CH_2_CH_2_N^+^(CH_3_)_3_Cl^−^.

**Table 4:** Comparison table of mass-to-charge ratio of gas produced via thermal decomposition of P(DAC-AM).

Decomposition Stage	*m*/*e*	Structures	*v*/cm^−1^
Stage I	17	NH_3_	3300~4000,1158
	18	H_2_O	3300~4000, 1626
	30	CH_3_CH_3_	2979, 2952
	41, 42	CH_2_=CH-CH_3_	1767~1560, 992
	44	CH_3_CHO, CH_3_CH_2_CH_3_	2979, 2952, 2823, 2774, 1735
	47	CH_3_CH_2_OH	1033–1053
	50, 52	CH_3_Cl	709
	59	N(CH_3_)_3_	1367
Stage II	17	NH_3_	3300~4000, 1158
	18	H_2_O	3300~4000, 1626
	30	CH_3_CH_3_	2979, 2952
	40	CH≡C-CH_3_	2180~2105
	41	CH_3_CN	2383~2305, 2180~2105
	42	CH_2_=CH-CH_3_	1767~1560, 992
	44	CO_2_, CH_3_CHO, CH_3_CH_2_CH_3_	2979, 2952, 2823, 2774, 2383~2305,1735, 671

**Table 5:** Initial apparent activation energy (*E*_(I)_ and *E*_(II)_) in Stage I and II.

Cationicity/%	0	10	30	50	70	90	100
*E*_(I)_/(kJ·mol^−1^)	174.16	196.93	180.36	193.64	216.09	220.51	221.32
*E*_(II)_/(kJ·mol^−1^)	236.63	220.34	210.16	211.28	201.53	218.69	237.18

## Data Availability

The original contributions presented in the study are included in the article/Supplementary Materials, further inquiries can be directed to the corresponding author.

## Supplementary Materials

The following supporting information can be downloaded at: https://www.mdpi.com/article/10.3390/polym16111522/s1, Figure S1. TG-DSC of P(DAC-AM), PAM and PDAC samples; Figure S2. Fitting curve of kinetics of P(DAC-AM), PAM and PDAC calculated using FWO method; Figure S3. Fitting curves of thermal decomposition kinetic parameters of P(DAC-AM), PAM and PDAC calculated using CR method; Table S1. Thermodynamic parameters of P(DAC-AM), PAM and PDAC samples; Table S2. Thermodynamic parameters of P(DAC-AM) with serial [*η*] and the same cationicity; Table S3. Thermal decomposition kinetic parameters of PAM and PDAC samples calculated using FWO method; Table S4. Thermal decomposition kinetic parameters of P(DAC-AM) with cationicity from 10% to 90%, [*η*] of 10 dL/g calculated using FWO method; Table S5. Thermal decomposition kinetic parameters of P(DAC-AM) with cationicity of 50%, [*η*] from 5 dL/g to 15 dL/g calculated using FWO method; Table S6. *r*^2^ of the fitting curves corresponding to different values of *n*; Table S7. *A* of P(DAC-AM), PAM and PDAC samples obtained from CR method.
