# Supplementary material for: Thermal Decomposition Mechanism of P(DAC-AM) with Serial Cationicity and Intrinsic Viscosity
Source: Polymers (Basel). 2024 May 28;16(11):1522. doi: 10.3390/polym16111522 (PMC11174957; doi:10.3390/polym16111522)
Supplement: Supplementary file 1 [file polymers-16-01522-s001.zip › polymers-2956038-supplementary.pdf]

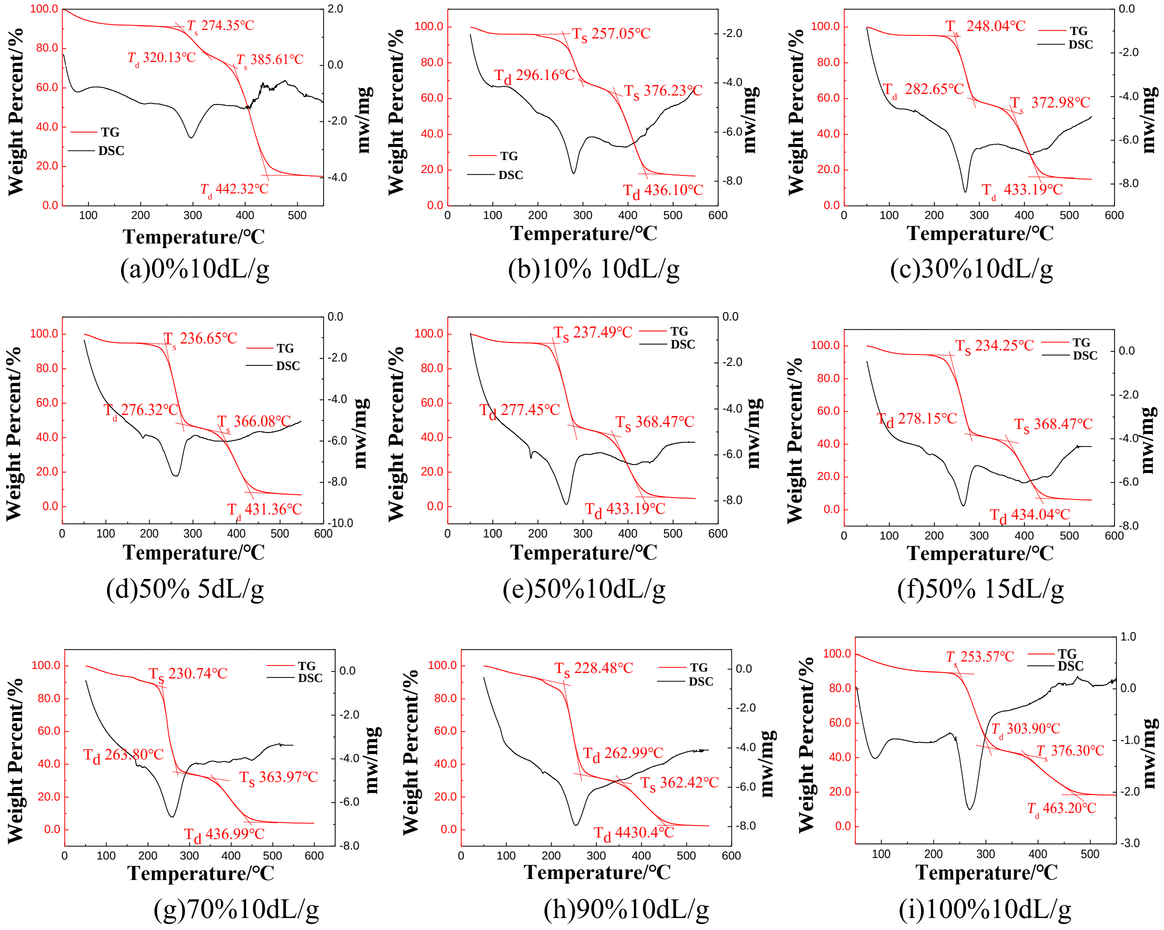

Figure S1. TG-DSC of P(DAC-AM), PAM and PDAC samples.

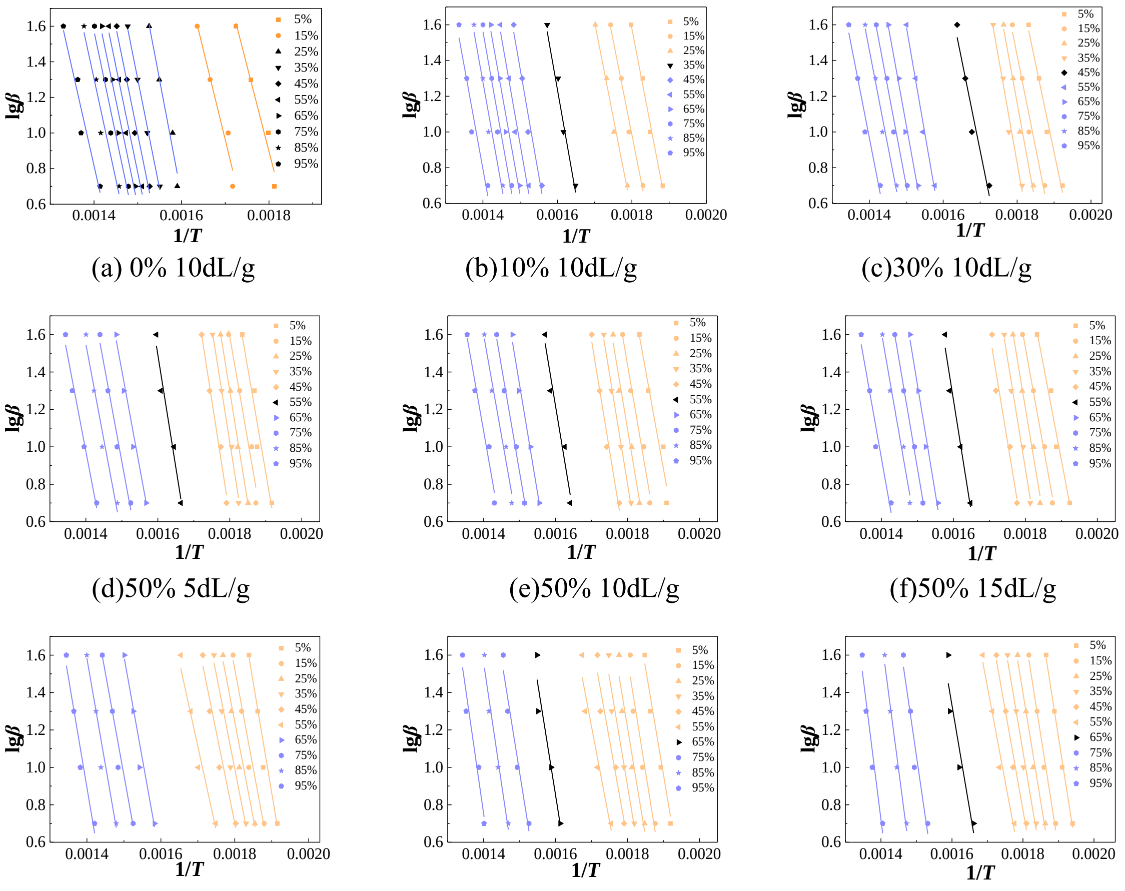

(a) 0% 10dL/g (b)10% 10dL/g (c)30% 10dL/g

(d)50% 5dL/g (e)50% 10dL/g (f)50% 15dL/g

(g)70% 10dL/g (h)90% 10dL/g (i)100% 10dL/g

Figure S2. Fitting curve of kinetics of P(DAC-AM), PAM and PDAC calculated by FWO method.

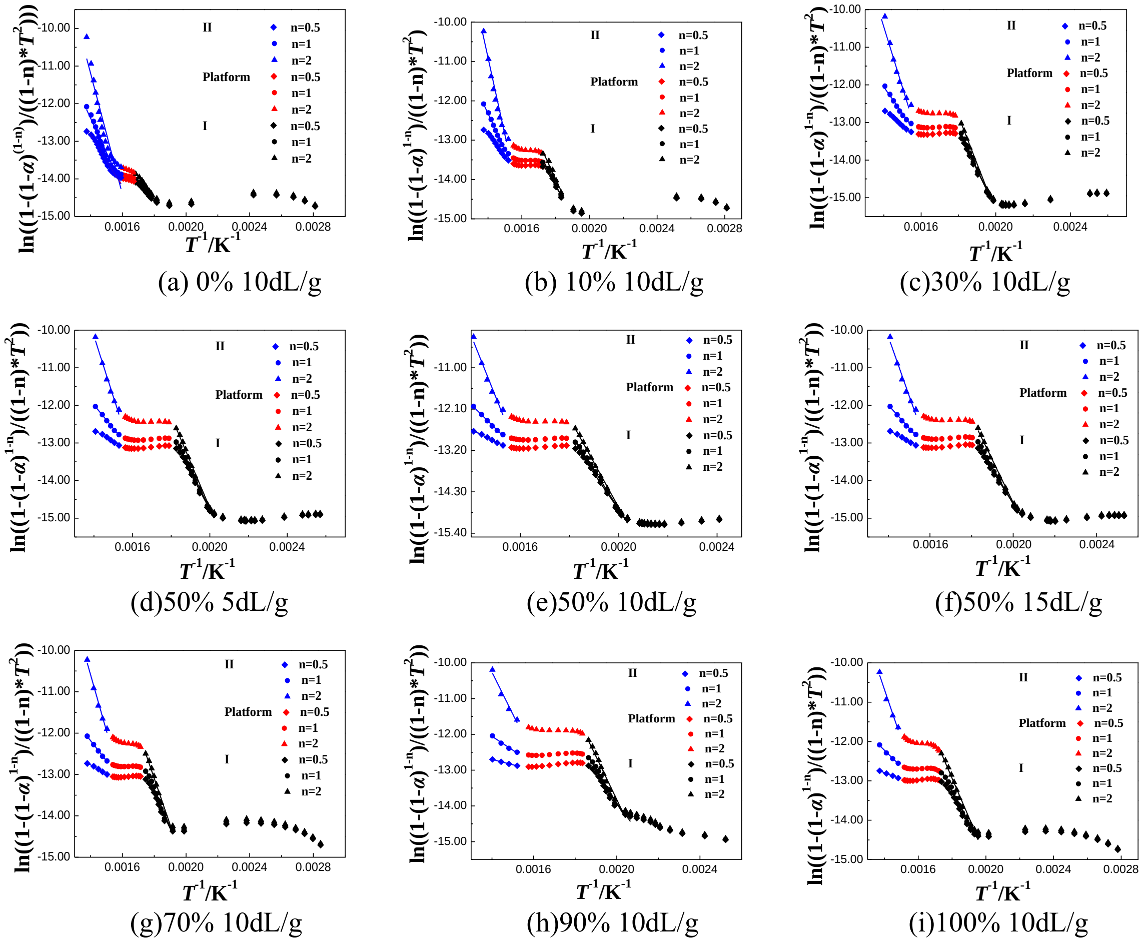

Figure S3. Fitting curves of thermal decomposition kinetic parameters of P(DAC-AM), PAM and PDAC calculated by CR method.

Table S1. Thermodynamic parameters of P(DAC-AM), PAM and PDAC samples

Cationicity/%	Stage I				Stage II			
	T_s/℃	T_d/℃	ΔH/(J•g^{-1})	W/%	T_s/℃	T_d/℃	ΔH/(J•g^{-1})	W/%
0	**274.35**	**320.13**	**198.04**	**17.48**	**385.61**	**441.13**	**149.24**	**66.02**
10	257.05	296.16	326.90	30.26	376.23	436.10	287.02	52.05
30	248.04	282.65	399.46	40.27	372.98	433.19	268.48	43.96
50	237.49	277.45	437.75	53.10	368.47	433.19	248.51	41.76
70	230.74	263.80	483.22	62.55	363.97	436.99	114.56	32.83
90	228.48	262.99	507.42	66.11	362.42	443.04	47.20	31.27
100	**253.57**	**303.90**	**130.91**	**51.31**	**376.30**	**463.20**	**17.19**	**28.47**

Table S2. Thermodynamic parameters of P(DAC-AM) with serial [η] and the same cationicity

[η]/ (dL•g^{-1})	Stage I				Stage II			
	T_s/℃	T_d/℃	ΔH/(J•g^{-1})	W/%	T_s/℃	T_d/℃	ΔH/(J•g^{-1})	W/%
5	236.65	276.32	448.17	51.72	366.08	431.36	288.34	41.12
10	237.49	277.45	437.75	53.10	368.47	433.19	248.51	41.76
15	234.25	278.15	**368.48**	53.24	368.47	434.04	**170.01**	40.61

Table S3. Thermal decomposition kinetic parameters of PAM and PDAC samples calculated by FWO method

α/%	E/(kJ•mol^{-1}) /r^2	
	PAM	PDAC
5	174.16/0.9487	221.32/0.9933
15	183.73/0.9348	219.17/0.9794
25	236.63/0.9550	211.25/0.9779
35	224.24/0.9941	204.86/0.9786
45	219.87/0.9745	195.00/0.9775
55	217.47/0.9561	183.96/0.9603
65	214.95/0.9396	**193.65/0.9236**
75	213.02/0.9312	237.18/0.9111
85	206.54/0.9242	263.36/0.9732
95	198.07/0.9102	274.08/0.9317

Table S4. Thermal decomposition kinetic parameters of P(DAC-AM) with cationicity from 10% to 90%, [η] of 10 dL/g calculated by FWO method

α/%	cationicity/%				
	10	30	50	70	90
	E/(kJ•mol^{-1}) /r^2				
5	196.93/0.9852	180.36/0.9702	193.64/0.9278	216.09/0.9498	220.51/0.9770
15	191.13/0.9793	188.19/0.9549	204.16/0.9705	195.18/0.9537	217.78/0.9032
25	193.96/0.9813	206.26/0.9719	210.15/0.9716	188.80/0.9540	212.66/0.9088
35	220.34/0.9704	213.23/0.9667	213.67/0.9938	186.41/0.9530	209.52/0.9230
45	217.26/0.9530	210.16/0.9963	213.93/0.9721	179.45/0.9420	194.85/0.9180
55	217.12/0.9529	217.03/0.9844	211.28/0.9432	173.56/0.9488	183.99/0.9025
65	212.97/0.9536	207.47/0.9796	210.53/0.9827	201.53/0.9483	213.75/0.9167
75	213.70/0.9520	199.46/0.9764	203.98/0.9875	199.37/0.9519	218.69/0.9473
85	212.36/0.9474	194.83/0.9706	197.62/0.9611	206.81/0.9324	236.44/0.9627
95	208.68/0.9085	192.59/0.9587	198.80/0.9576	213.45/0.9470	242.63/0.9332

Table S5. Thermal decomposition kinetic parameters of P(DAC-AM) with cationicity of 50%, [η] from 5 dL/g to 15 dL/g calculated by FWO method

α/%	[η]/(dL•g^{-1})		
	5	10	15
	E/(kJ•mol^{-1}) /r^2		
5	203.34/0.9159	193.64/0.9278	184.73/0.9582
15	202.30/0.9480	204.16/0.9705	193.54/0.9909
25	217.14/0.9946	210.15/0.9716	210.41/0.9861
35	222.03/0.9924	213.67/0.9938	215.79/0.9821
45	225.56/0.9785	213.93/0.9721	212.15/0.9374
55	219.18/0.9507	211.28/0.9432	220.16/0.9601
65	195.52/0.9769	210.53/0.9827	212.42/0.9783
75	190.52/0.9754	203.98/0.9875	206.32/0.9965
85	186.33/0.9530	197.62/0.9611	200.41/0.9698
95	183.25/0.9751	198.80/0.9576	196.95/0.9400

Table S6. r^2 of the fitting curves corresponding to different values of n

No.	P(DAC-AM)		r^2					
	cationicity/%	$[\eta]$/(dL•g^{-1})	I			II		
			n=0.5	n=1	n=2	n=0.5	n=1	n=2
1	0		0.9891	0.9894	0.9893	0.9711	0.9756	0.9119
2	10		0.9516	0.9978	0.9663	0.9883	0.9905	0.9598
3	30		0.9946	0.9976	0.9918	0.9801	0.9824	0.9448
4	50	10	0.9950	0.9956	0.9943	0.9942	0.9944	0.9718
5	70		0.9779	0.9781	0.9746	0.9920	0.9973	0.9799
6	90		0.9694	0.9779	0.9710	0.9749	0.9778	0.9539
7	100		0.9853	0.9859	0.9829	0.9803	0.9830	0.9544
8		5	0.9931	0.9963	0.9802	0.9867	0.9931	0.9906
9	50	10	0.9950	0.9956	0.9943	0.9942	0.9944	0.9718
10		15	0.9952	0.9981	0.9933	0.9904	0.9935	0.9697

Table S7. A of P(DAC-AM), PAM and PDAC samples obtained from CR method

No.	P(DAC-AM)		A/s^{-1}	
	cationicity/%	$[\eta]$/(dL•g^{-1})	I	II
1	0		1.89×10^{1}	9.91×10^{2}
2	10		5.84×10^{3}	6.54×10^{2}
3	30		8.98×10^{5}	1.91×10^{2}
4	50	10	6.12×10^{5}	3.66×10^{1}
5	70		3.16×10^{4}	1.76×10^{0}
6	90		1.08×10^{4}	8.40×10^{-1}
7	100		4.37×10^{3}	7.10×10^{-1}
8		5	2.73×10^{6}	4.28×10^{1}
9	50	10	6.12×10^{5}	3.66×10^{1}
10		15	1.41×10^{5}	3.30×10^{1}
